# Fibronectin supports neurite outgrowth and axonal regeneration of adult brain neurons in vitro

**DOI:** 10.1016/j.brainres.2012.03.024

**Published:** 2012-05-09

**Authors:** David A. Tonge, Hugo T. de Burgh, Reginald Docherty, Martin J. Humphries, Susan E. Craig, John Pizzey

**Affiliations:** a Wolfson-Centre for Age-Related Disease, King's College London, Hodgkin Building, Guy's Campus, London SE1 1UL, UK; b Wellcome Trust Centre for Cell-Matrix Research, School of Biological Sciences, University of Manchester, Michael Smith Building, Oxford Road, Manchester M13 9PT, UK

**Keywords:** Axon, Regeneration, Hippocampus, Cortex, Fibronectin

## Abstract

The molecular basis of axonal regeneration of central nervous system (CNS) neurons remains to be fully elucidated. In part, this is due to the difficulty in maintaining CNS neurons in vitro. Here, we show that dissociated neurons from the cerebral cortex and hippocampus of adult mice may be maintained in culture for up to 9 days in defined medium without added growth factors. Outgrowth of neurites including axons was observed from both CNS sources and was significantly greater on plasma fibronectin than on other substrata such as laminin and merosin. Neurite outgrowth on fibronectin appears to be mediated by α5β1 integrin since a recombinant fibronectin fragment containing binding sites for this receptor was as effective as intact fibronectin in supporting neurite outgrowth. Conversely, function-blocking antibodies to α5 and β1 integrin sub-units inhibited neurite outgrowth on intact fibronectin. These results suggest that the axonal regeneration seen in in vivo studies using fibronectin-based matrices is due to the molecule itself and not a consequence of secondary events such as cellular infiltration. They also indicate the domains of fibronectin that may be responsible for eliciting this response.

## Highlights

► Fibronectin as a substratum for adult mammalian neurons. ► Increased neurite growth from hippocampal and cortical neurons grown on fibronectin. ► Increased neurite growth on fibronectin appears to involve α5β1 integrin.

## 1 Introduction

The failure of central nervous system (CNS) axons to regenerate following injury is widely believed to be due to a combination of the presence of inhibitory factors, lack of trophic support and also intrinsically poor capacity for regeneration (Sun and He, 2010). However, CNS axons can regenerate for long distances in peripheral nerve grafts (see Ferguson et al., 2001 and references therein), indicating that they are innately capable of regeneration in a permissive environment and that failure of axonal regeneration in the CNS may be partly due to lack of factor(s) required to support axonal growth. Retinal ganglion cells of adult mammals show enhanced axonal sprouting and/or regeneration in response to factors such as oncomodulin (Yin et al., 2006), osteonectin (Ma et al., 2010), cell adhesion molecules (Zhang et al., 2008) and neurotrophic factors (reviewed by Cui, 2006) but there have been few studies involving neurons from the brains of adult animals (Brewer, 1997, Brewer and Torricelli, 2007, Nathan et al., 2002, Richter and Roskams, 2009) and consequently their growth requirements are largely unknown.

Fibronectin (FN) is a large glycoprotein present in plasma and also an important component of the extracellular matrix (ECM). FN usually comprises two similar 250 kDa subunits linked near their C-termini by disulfide bonds, with each subunit containing 3 types of repeating module (types I, II and III). FN is known to act as a ligand for at least 11 different integrin heterodimers (Leiss et al., 2008) supporting adhesion of numerous cell types and also neurite outgrowth from developing peripheral nervous system (PNS) and central nervous system (CNS) neurons (Akers et al., 1981, Carri et al., 1988, Haugen et al., 1992, Humphries et al., 1988, Rogers et al., 1985, Rogers et al., 1988). In adult animals, FN is up-regulated in lesioned peripheral nerves (Lefcort et al., 1992, Siironen et al., 1992) and α5β1 integrin, an important FN receptor, is expressed on regenerating axons (Lefcort et al., 1992, Yanagida et al., 1999) and mediates neurite outgrowth from dorsal root ganglion (DRG) neurons on FN in vitro (Gardiner et al., 2007). In the CNS, α5β1 integrin is expressed by neurons during development and although down-regulated during maturation (Yoshida et al., 2003) is still detectable in the brains of mature animals (King et al., 2001) including neurons in the hippocampus and cerebral cortex (Bi et al., 2001). The observations above suggest that FN might thus be capable of supporting axonal regeneration in the CNS of adult animals. Consistent with this hypothesis, FN mats have been used to bridge gaps in injured spinal cords of adult rats, into which axonal regeneration then occurred (King et al., 2003, Taylor et al., 2004). However, in these experiments axonal growth was associated with cellular infiltration and it is therefore uncertain whether FN directly supports axonal regeneration. In mature animals, FN is present in some axonal tracts in the CNS and can mediate axonal growth from dissociated DRG neurons on brain slices in culture, but only dendritic outgrowth was observed from dissociated cortical neurons (Tom et al., 2004).

In the present investigations, we demonstrate that cultured cortical and hippocampal neurons from adult mice show robust neurite and axonal outgrowth on FN which is associated with expression of markers associated with axonal regeneration, including growth-associated protein 43 kDa (GAP-43) and phosphorylated ribosomal protein S6 (p-S6) a marker of mTOR activation (Caroni, 1997, Christie et al., 2010, Liu et al., 2010). Neurite outgrowth on FN is mainly mediated by α5β1 integrin and consistent with these findings, a recombinant 50 kDa FN fragment (FN50K) containing binding sites for this receptor is highly effective in supporting axonal growth of dissociated hippocampal neurons. Our results suggest that FN might be a potential substrate for CNS axonal regeneration in vivo.

## 2 Results

### 2.1 Characterization of cortical and hippocampal cultures

**Fig. 1:**
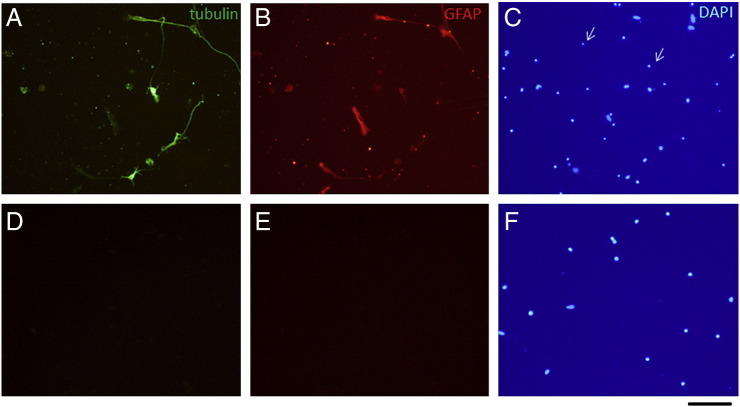
Dissociated hippocampal cells after 3 days in culture contain both βIII tubulin-positive neurons and GFAP-positive astrocytes. Among the cell nuclei labeled with DAPI are some small pyknotic nuclei of dead cells (arrows in C; see also Supplementary Fig. 1) which are distinguishable from the larger nuclei of live cells and were therefore excluded from the total cell counts. D and E show absence of labeling of cells, whose nuclei can be seen in (F), when the primary antibodies were omitted. Scale bar = 100 μm. Dissociated hippocampal cells after 3 days in culture contain both βIII tubulin-positive neurons and GFAP-positive astrocytes. Among the cell nuclei labeled with DAPI are some small pyknotic nuclei of dead cells (arrows in C; see also Supplementary Fig. 1) which are distinguishable from the larger nuclei of live cells and were therefore excluded from the total cell counts. D and E show absence of labeling of cells, whose nuclei can be seen in (F), when the primary antibodies were omitted. Scale bar = 100 μm.

Preparations of cultured dissociated adult CNS neurons are difficult to establish. It was therefore essential initially to confirm that our cultures contained significant numbers of neurons and that both axons and neurites could be identified. To achieve this we dissociated cells from the hippocampus and cortex of adult mice and cultured them in multiwells coated with FN for 3 days (Fig. 1). They were then fixed, followed by immunocytology using antibodies to βIII tubulin and glial fibrillary acidic protein (GFAP) to estimate the percentages of neurons and astrocytes as proportions of total cell populations determined from DAPI-labeled nuclei. Results from 3 experiments showed that in hippocampal and cortical cultures 37 ± 4% (of 329 cells counted) and 21 ± 6% of cells (of 458 cells counted), respectively were neurons on the basis of βIII tubulin expression. The proportions of astrocytes in hippocampal and cortical cultures, identified by expression of GFAP, were much lower (9 ± 5% and 12 ± 7%, respectively). In the CNS, βIII tubulin is widely used as a marker of immature neurons (Menezes and Luskin, 1994) but is down-regulated in adult animals (Jiang and Oblinger, 1992) and it is therefore possible that the proportions of neurons in our cultures might be underestimated by using antibodies to this marker. We therefore additionally compared labeling of cultures using antibodies to βIII tubulin and protein gene product 9.5 kDa (PGP 9.5), also known as ubiquitin carboxyl-terminal hydrolase-1, a protein expressed by almost all neurons (reviewed by Day and Thompson, 2010) and observed that both antibodies labeled identical cells in both hippocampal and cortical cultures (data not shown). However, the antibody to PGP 9.5 tended to give stronger and more even labeling of neurites and axons, and was therefore used routinely for quantification of neurite/axonal growth. In some initial experiments NE14, an antibody to heavy neurofilament (NF200) was used to label neurons and their processes. However later experiments included antibodies to βIII tubulin or PGP 9.5 since they had the same specificities as NE14 but gave stronger immunostaining.

**Fig. 2:**
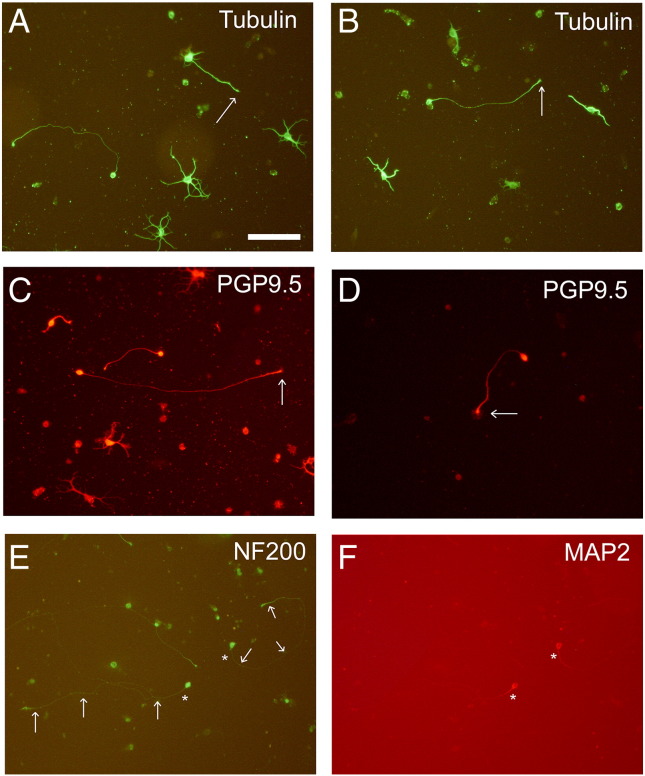
Extension of neurites, including axons on FN after 3 days in culture. Dissociated hippocampal (A and C) and cortical cells (B and D) labeled using antibody to βIII tubulin (A and B) showing neurons with neurites and also some axons terminating in growth cones (arrows), which are more clearly visible when labeled with antibody to PGP 9.5 (C and D). A hippocampal cell culture labeled with antibody to NF200 (E) showing neurons (asterisks) with uniform labeling along the length of the axon (arrows). Distal parts of the same axon are either faintly labeled or unlabeled with an antibody to MAP2 (F) where only the cell bodies and proximal axonal segments react strongly. Scale bar = 100 μm.

In cultures of both hippocampal and cortical cells, approximately half the neurons (Table 2) extended processes, often comprising several tapering neurites. While most of the neurons extended processes like these, a smaller proportion extended a single axon which often ended in a distinct growth cone (Fig. 2). In addition to the presence of a single process and apparent growth cone, axons could also be distinguished by the absence of microtubule associated protein 2 (MAP2) in cultures of both cortical and hippocampal cells (Figs. 2E and F). MAP2 is a marker for dendrites (de Lima et al., 1997, Dotti et al., 1988, Kanai and Hirokawa, 1995) but is absent from CNS axons apart from the axon hillock and initial segment (Bernhardt and Matus, 1984). We further confirmed the use of MAP2 in axonal identification in a series of experiments in which we cultured neurons for 6 days instead of 3 days (data not shown). When axons form in vitro, they initially express MAP2, but this is lost (except for the hillock and initial segment) after 4–5 days (Dotti et al., 1988).

**Table 1:** Primary antibodies used for immunocytology and cell culture.

Antigen	Host/type (clone)	Dilution	Source
βIII tubulin	Rabbit polyclonal	1:1000	Abcam
PGP 9.5	Rabbit polyclonal	1:1000	Biogenesis
GFAP	Rabbit polyclonal	1:1000	Dako
GAP 43	Rabbit polyclonal	1:500	Chemicon
NF200	Mouse, mAb (NE 14)	1:200	Sigma
p-S6	Rabbit polyclonal	1:200	Cell Signaling Technology Inc
α5 integrin	Rat mAb (5H10)	20 μg/ml	Serotec
β1 integrin	Hamster mAb (Ha2/5)	10 μg/ml	Serotec

**Table 2:** The mean proportions of neurons (± SEM) with neurites or axons in cultures of hippocampal and cortical cells on FN after 3 days. Data represent the mean (± SEM) of 188 hippocampal and 165 cortical neurons measured.

Hippocampus		Cortex	
Neurites	Axons	Neurites	Axons
50 ± 5%	30 + 2%	55 + 6%	16 + 3%

**Fig. 3:**
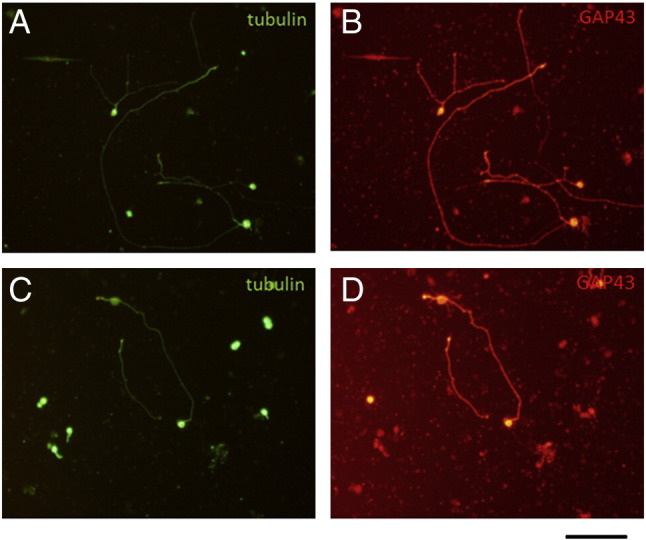
Hippocampal (A and B) and cortical (C and D) cell cultures showing co-expression of βIII tubulin and GAP43 in neurons. Scale bar = 100 μm.

**Fig. 4:**
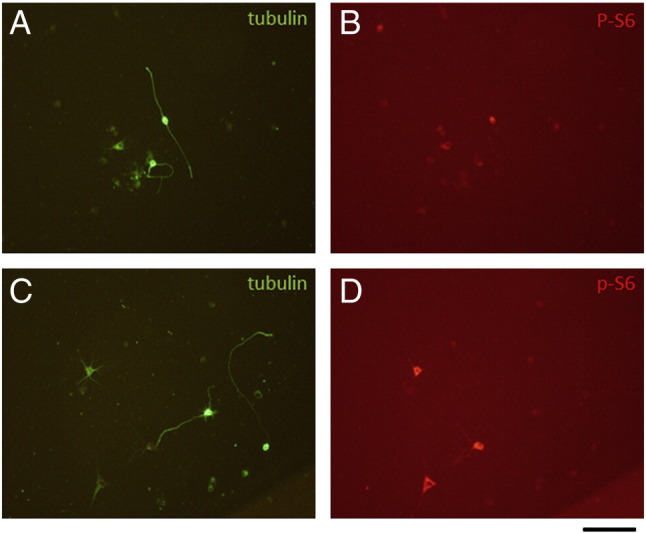
Lack of correlation between p-S6 immunoreactivity and neurite outgrowth. Hippocampal cultures showing association of p-S6 labeling with neurite outgrowth (A and B) and lack of correlation (C and D). Scale bar = 100 μm.

During axonal regeneration in the PNS and CNS in vivo, neurons express a number of regeneration-associated genes (RAGs; reviewed by Rossi et al., 2007) of which the best characterized is growth-associated protein 43 kDa (GAP 43). In 3 day cultures of hippocampal and cortical cells, almost all neurons showed strong expression of GAP 43 (Fig. 3), indicating spontaneous expression of RAGs which might explain their vigorous outgrowth of neurites and axons. Axonal regeneration in vivo is also associated with phosphorylation of ribosomal protein S6 (p-S6) a marker of mTOR activation (Christie et al., 2010, Liu et al., 2010). In hippocampal and cortical cell cultures, p-S6 immunoreactivity was present in both neurons and also non-neuronal cells. However, many neurons showed neurite or axonal outgrowth without p-S6 immunoreactivity (Fig. 4), indeed there was no obvious correlation between process outgrowth and p-S6 immunolabeling which may suggest that mTOR activation is not essential for process outgrowth.

### 2.2 Fibronectin promotes neurite outgrowth of adult CNS neurons in vitro

**Fig. 5:**
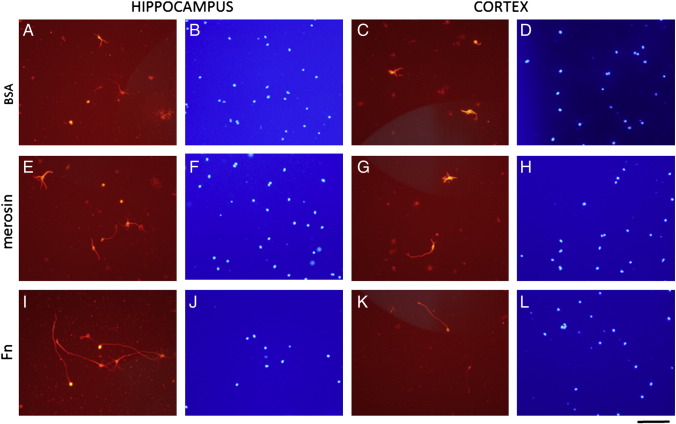
Mouse hippocampal and cortical neurons (visualized by labeling for PGP 9.5) and nuclei of all cells (visualized by DAPI labeling) after 3 days culture on BSA (A, B, C, and D) merosin (E, F, G, and H) and FN (I, J, K, and L). Lengths of neurites (including axons) are greater on FN than BSA or merosin. Scale bar = 100 μm.

To test the hypothesis that FN alone is capable of supporting neurite outgrowth from adult CNS neurons we compared the effectiveness of FN with other substrata in supporting neurite outgrowth, mouse hippocampal and cortical cells were cultured in multiwells coated with a variety of substrata. In addition to an FN substratum, wells were also coated with BSA, laminin, poly-l-lysine, merosin or uncoated tissue culture plastic was used. Further, we assayed for neurite outgrowth with adult guinea pig CNS neurons (data not shown) in addition to hippocampal and cortical neurons from adult mice. After 3 days, preparations were incubated with calcein to label all live cells, followed by fixing and labeling with antibody to PGP 9.5 to identify neurons and their processes. In all cases, FN proved to be the most effective substratum for CNS neurons and results for BSA, merosin and FN are shown in Fig. 5 and Table 3. Results of these experiments showed that although there were no significant differences in the proportions of neurons extending neurites on these substrates, neurite lengths were significantly greater (P < 0.05) on FN compared to BSA or merosin in both cortical and hippocampal cultures (Table 3). Importantly, visualization of live cells with calcein showed that neurite outgrowth had occurred on the underlying substrata and not over non-neuronal cells (Supplementary Fig. 2).

**Table 3:** The mean percentages (± SEM) of neurons and neurite lengths in dissociated cultures of cells from mouse hippocampus and cortex cultured for 3 days on different substrata. The mean proportions of neurons extending processes on the different substrata were not significantly different but the mean lengths of the longest neurites were significantly greater on FN than BSA or merosin. The differences in neurite lengths on BSA and merosin were not significantly different. Numbers in parentheses beside each substratum indicate the total number of viable cells counted. * Indicates significant difference from corresponding values for BSA or merosin at P < 0.05.

	Hippocampus			Cortex		
Substratum	BSA (285)	FN (274)	Merosin (308)	BSA (263)	FN (357)	Merosin (280)
Neurons as % of viable cells	31 (± 3)	39 (± 6)	34 (± 7)	36 (± 5)	45 (± 4)	36 (± 2)
% of neurons with neurites	36 (± 6)	64 (± 16)	61 (± 20)	25 (± 7)	38 (± 9)	29 (± 6)
Mean neurite length in μm	62 (± 13)	157 (± 16)*	74 (± 11)	48 (± 4)	131 (± 22)*	54 (± 7)

### 2.3 A 50K fragment of fibronectin is sufficient to produce neurite outgrowth from CNS neurons in vitro

**Fig. 6:**
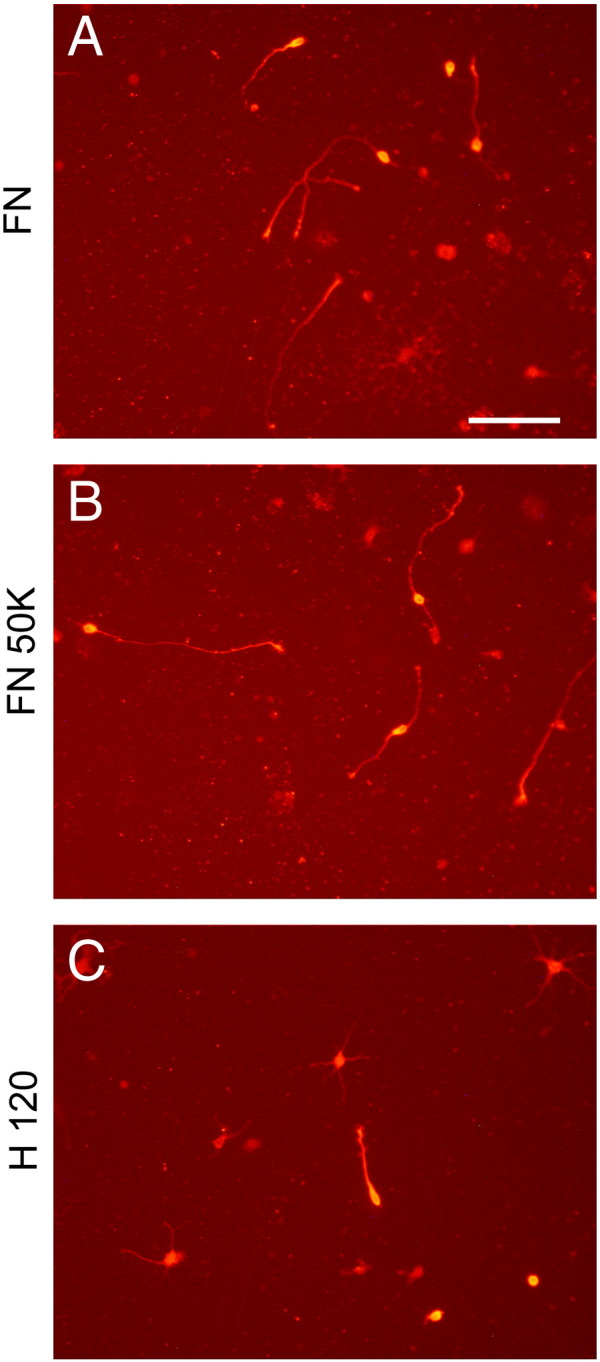
Dissociated mouse hippocampal neurons, labeled for PGP 9.5, after 3 days culture on FN (A) and recombinant FN fragments FN50K (B) and H120 (C). Neurite lengths are greater on FN and FN50K than on H120. Scale bar = 100 μm.

Having established that a FN substratum can support neurite outgrowth from adult CNS neurons, we then performed experiments to investigate which functional domains of the FN molecule might be effecting this response. Neurite outgrowth from DRG neurons on FN is mediated principally by α5β1 integrin (Gardiner et al., 2007). However, α4β1 integrin mediates neurite outgrowth of sympathetic neurons and also PC12 cells on FN (Vogelezang et al., 2007, Wingerd et al., 2005). To investigate the possible involvement of these integrins in neurite outgrowth, hippocampal cells were cultured on recombinant FN fragments 50 K containing the CCCB domain (including α5β1 integrin binding site) and H120 (with α4β1 integrin binding site). Results clearly showed that the FN50K fragment supported excellent neurite and axonal growth but the H120 fragment did not (Fig. 6, Table 4), suggesting dependence on α5β1 integrin rather than α4β1 integrin. However, the FN50K fragment also contains binding sites for the αv integrin family and thus it cannot be excluded that these may have additional roles in neurite outgrowth and axonal regeneration.

**Table 4:** Mean lengths (± SEM) of neurites from mouse hippocampal neurons after 3 days in culture on FN or recombinant FN fragments. Axonal lengths on FN and FN 50 kDa were significantly (P < 0.02) greater than on the H120 fragment but the difference in outgrowth lengths on FN and FN 50 kDa was not significant. Numbers in parentheses beside each substratum indicate the total number of viable cells counted.

Substratum	FN (34)	FN 50 kDa (32)	H120 (44)
Mean neurite length	145 ± 20 μm	226 ± 39 μm	62 ± 4 μm

**Fig. 7:**
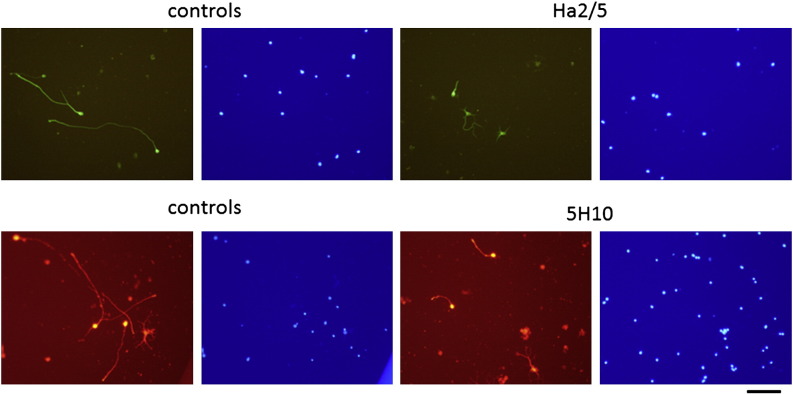
Dissociated mouse hippocampal neurons, labeled for β1II tubulin (upper panels) or PGP 9.5 (lower panels), after 3 days culture on FN in the presence of antibodies to β1 integrin (Ha2/5) or α5 integrin (5H10) compared with control cultures without antibodies. Scale bar = 100 μm.

To determine directly whether α5β1 integrin mediates axonal growth from CNS neurons on FN, a function-blocking antibody to β1 integrin (Ha2/5) was added to neuronal cultures (Fig. 7). In hippocampal preparations cultured with 10 μg/ml of Ha2/5 for 3 days, the mean length of outgrowing neurites was 78 ± 11 μm (62 neurons counted), which was significantly less (P < 0.05) than in control preparations cultured but without the antibody (170 ± 26 μm; 67 neurons counted). The proportion of neurons (as a percentage of DAPI-labeled nuclei) on FN and FN50K were similar to other experiments described here but the proportion on H120 appeared much lower. This probably reflects the inability of neurons to adhere to this substratum or its ineffectiveness in their subsequent survival.

In further experiments, the mean neurite length of hippocampal neurons on FN in the presence of a blocking antibody to α5 integrin (5H10) was 81 ± 9 μm (46 neurons counted), which was also significantly less (P < 0.02) compared to that in control cultures without the antibody (159 ± 17 μm; 44 neurons counted), consistent with an involvement of α5β1 integrin.

## 3 Discussion

Results of the present investigations demonstrate that neurons from the brains of adult mice survive for several days in culture, confirming earlier findings (Brewer, 1997, Brewer and Torricelli, 2007, Nathan et al., 2002). The cultures contained both glutamatergic and GABAergic neurons (data not shown) and in all experiments, a surprisingly high proportion of neurons (typically 30–60%) extended neurites, even on plastic or BSA. However, we also show that neurite lengths were significantly greater on FN than on other substrata tested and that in cultures of both hippocampal and cortical cells, many neurons extended axons (Table 2). These findings indicate that such cultures could be used to identify factors supporting the growth of different populations of CNS neurons.

It is well established that FN supports neurite outgrowth from developing CNS and PNS neurons (Carri et al., 1988, Haugen et al., 1992, Humphries et al., 1988, Rogers et al., 1985, Rogers et al., 1988). Moreover, the ability of different domains of the FN molecule to support neurite outgrowth from developing neurons has been extensively investigated using proteolytic fragments generated by sequential digestion with trypsin and cathepsin D, followed by chromatography. Results of such studies (Carri et al., 1988, Haugen et al., 1992, Rogers et al., 1985, Rogers et al., 1988) have shown that fragments containing the main heparin-binding domain of the FN molecule, situated in type III repeat 13, support neurite outgrowth from chick spinal cord and retina whereas fragments containing the CCBD, located in type III repeat 10, are less effective. In view of these studies, hippocampal cells from adult mice were cultured on recombinant FN fragments FN50K, containing the CCBD with binding sites for integrins α5β1 and αvβ3 and H120, containing the α4β1 integrin binding site and the main heparin-binding domain (Mostafavi-Pour et al., 2003). Our results using dissociated cell cultures from brains of adult mice show that the FN50K fragment containing the CCBD domain strongly supports axonal growth from hippocampal neurons whilst the H120 fragment does not, contrary to what might have been expected from previous studies using CNS neurons from immature animals. The ability of the FN50K fragment to support neurite outgrowth suggests involvement of α5β1 integrin and consistent with this result, antibodies to both α5 and β1 integrin sub-units significantly reduced neurite outgrowth on intact FN. Interestingly, forced expression of α5 integrin promotes neurite outgrowth of both DRG neurons and also NT2N CNS neuronal cells (Condic, 2001, Meland et al., 2010).

The relative ineffectiveness of the H120 fragment in supporting neurite outgrowth in our experiments is surprising given that fragments containing the heparin-binding domain support neurite outgrowth from embryonic CNS neurons. The H120 fragment contains binding sites for α4β1 integrin this integrin mediates neurite outgrowth from PC12 cells, sympathetic neurons and retinal ganglion cells (Hikita et al., 2003, Vogelezang et al., 2007, Wingerd et al., 2005). However α4β1 integrin does not appear to be involved in mediation of neurite outgrowth of dorsal root ganglion neurons on FN (Gardiner et al., 2007). Interestingly, expression of α4β1 integrin is sharply reduced in the CNS during post-natal development (Milner and Campbell, 2002), which might account for relative ineffectiveness of the H120 FN fragment in supporting neurite outgrowth.

Little is known about the ability of FN to support axonal regeneration in the CNS although Tom et al. (2004) demonstrated that FN in CNS white matter could promote axonal growth of dissociated DRG neurons on cultured forebrain slices. In their experiments, they also observed neurite outgrowth from dissociated cortical neurons but classified these processes as dendrites rather than axons on the basis of MAP2 immunoreactivity. The presence of FN in CNS white matter might help to explain why axonal regeneration can occur within adult CNS pathways (Davies et al., 1999), providing that glial scarring is absent. FN is present at high levels in plasma, but is normally excluded from the CNS except transiently during extravasation following injury (Liu and Sturner, 1988). It is possible that ‘abortive’ axonal sprouting often observed during the first weeks after CNS lesions (reviewed by Schwab and Bartholdi, 1996) may be sustained, partly, by the temporary presence of plasma FN adjacent to CNS lesion sites. Axonal regeneration does not occur in the mature CNS, although there is abundant initial sprouting after injury, which might be due to influx of plasma FN and also localized FN expression. This suggests that increased availability of FN might promote axonal regeneration in the CNS and is consistent with the findings of King et al. (2003) that FN mats support axonal ingrowth in the injured spinal cord.

## 4 Experimental procedures

All reagents were purchased from Sigma unless otherwise stated.

### 4.1 Cell cultures

Experiments were performed using tissue from CD-1 mice aged 3–6 weeks (bred at King's College London) and killed by deep anesthesia using Euthanal Pentobarbital sodium (Merial, Essex UK), followed by rapid removal of the hippocampi and cerebral cortex by dissection. For each experiment where neurite outgrowth under different conditions was compared, mice 3–6 weeks of age were used. However in each experiment, the same pool of neurons was used for each experimental condition.

Pairs of mouse hippocampi or similar sized sections of cerebral cortex, were minced with micro-scissors and transferred to loosely-capped universal tubes containing 1 ml solution of a solution of 20 units/ml papain in 1 mM l-cysteine and 0.5 mM EDTA and 0.005% DNAse (Worthington). The universal tubes were agitated at approximately 400 rpm in an atmosphere of 95% O_2_/5% CO_2_ for 90 min at room temperature, followed by trituration using plastic pipette tips. The suspensions of dissociated tissues were centrifuged at 150 ×*g* for 5 min and the pellet re-suspended in 2 ml of Earle's balanced salt solution (EBSS) which was then layered onto 1 ml of a solution of 20 mg/ml bovine serum albumin (BSA) in EBSS, followed by further centrifugation at 150 ×*g* for 5 min. The resulting pellet was re-suspended in 4 ml of Neurobasal-A with B27 supplement (Invitrogen) containing 2 mM glutamine, 100 U/ml penicillin, 100 μg/ml streptomycin and 250 ng/ml amphotericin B. Aliquots of 0.5 ml of the suspension were added to 4-well culture dishes (Nunc), coated for 1 h with solutions of 20 μg/ml of bovine serum albumin (BSA), laminin (BD Biosciences), merosin (Chemicon) or bovine plasma FN (Sigma), all in PBS. In 3 independent experiments, the initial numbers of live cells per well in hippocampal and cortical cultures were 38,500 ± 7000 and 56,500 ± 7000 respectively, estimated following incubation for 20 min with 1 μM Calcein AM (Invitrogen), a membrane permeable dye which is hydrolyzed by intracellular esterases in viable cells to yield a fluorescent green product. In some experiments, wells were coated with 20 μg/ml recombinant human FN fragments FN50K or H120 (Mostafavi-Pour et al., 2003). FN50K comprises FN type III repeats 6–10 inclusive, containing the central cell binding domain (CCBD) and binding sites for integrins αVβ3 and α5β1. The H120 fragment is a construct including type III repeats 12–15 containing the binding site for integrin α4β1.

### 4.2 Immunocytology

Cultures were fixed after 3 or 6 days for approximately 30 min with 3.6% formaldehyde in phosphate buffered saline (PBS), washed with PBS and then blocked with 3% (w/v) BSA,0.1% (v/v) Triton X-100 in PBS for a further 30 min. Preparations were incubated overnight at 4 °C with primary antibodies (Table 1) and next day washed in PBS, followed by incubation with Alexa 488- or Alexa 568-conjugated secondary antibodies (Invitrogen) for 1 h. After incubation with the second antibodies, preparations were washed with PBS prior to mounting in Vectashield containing 4′,6-diamidino-2-phenylindole (DAPI, Vector Laboratories, Burlingame, USA). In some experiments, cells were first labeled using 1 μg/ml calcein AM or calcein Orange-Red (Invitrogen), which are hydrolyzed in living cells to yield fluorescent green or red products, respectively. This was followed by formaldehyde fixation and subsequent immunocytochemistry as described above. This procedure also made it possible to identify the small pyknotic nuclei of dead cells (visualized by DAPI labeling) which were excluded from counts of total cell numbers.

Labeled cultures were viewed using an Eclipse TE200 fluorescence microscope (Nikon, Japan), and images captured using a DXM1200F digital camera (Nikon, Japan).

### 4.3 Stereography

Following immunocytology, 6 frames randomly taken along orthogonal diameters were captured for each well providing images of 100–200 cells for analysis. The numbers of neurons, identified by βIII tubulin or PGP 9.5 labeling, and the proportion with neurites (longer than 1 cell diameter) per image were counted, and neurite lengths were quantified from the digital images by measuring the longest neurite of all neurons in the frames, using a PC version of NIH Image (Scion Image). All experiments were repeated 3 times unless otherwise stated and experimental data are expressed as means ± S.E.M. The differences between means were evaluated by a Student's *t* test where appropriate and considered significant at P < 0.05.

The following are the supplementary materials related to this article.

**Supplementary Fig. 1:**
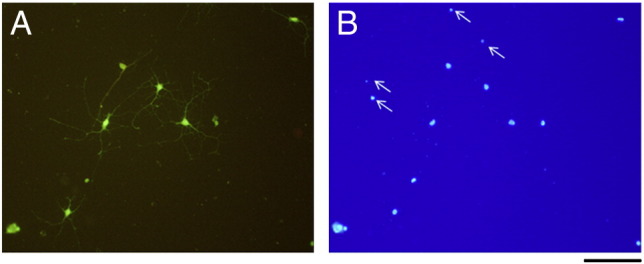
Hippocampal cell culture incubated with calcein AM (A) to label live cells (A) followed by fixing and mounting in Vectashield with DAPI to label nuclei (B). Note that some small nuclei (arrows in B) are not associated with calcein labeling and therefore correspond to dead cells. Scale bar = 100 μm.

**Supplementary Fig. 2:**
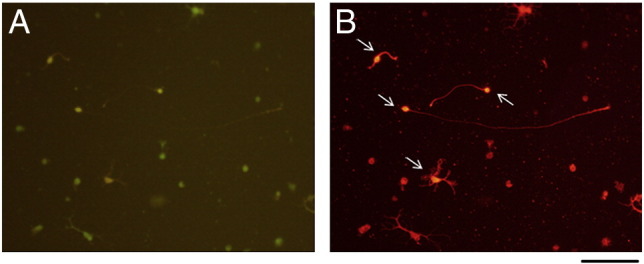
Photomicrographs of live hippocampal cells labeled with calcein (A) before fixing and staining for PGP 9.5 (B) to visualize neurons. Note that axons and neurites of the intensely-labeled neurons (arrows in B) extend on the substratum (FN) rather than on the processes of other cells, visualized by calcein labeling in A. Scale bar = 100 μm.
